# Integrating multi-platform genomic data using hierarchical Bayesian relevance vector machines

**DOI:** 10.1186/1687-4153-2013-9

**Published:** 2013-06-28

**Authors:** Sanvesh Srivastava, Wenyi Wang, Ganiraju Manyam, Carlos Ordonez, Veerabhadran Baladandayuthapani

**Affiliations:** 1 Department of Statistics, Purdue University, 250 N. University Street, West Lafayette, IN 47907, USA; 2 Department of Bioinformatics and Computational Biology, Division of Quantitative Sciences, The University of Texas MD Anderson Cancer Center, 1515 Holcombe Blvd, Unit 1411, Houston, Texas, USA; 3 Department of Computer Science, University of Houston, 4800 Calhoun, Houston, Texas, USA; 4 Department of Biostatistics, Division of Quantitative Sciences, The University of Texas MD Anderson Cancer Center, 1515 Holcombe Blvd, Unit 1411, Houston, Texas, USA

**Keywords:** Bayesian modeling; Multiple kernel learning; Genomics; High-dimensional data analysis; Prediction; Variational inference

## Abstract

**Background:**

Recent advances in genome technologies and the subsequent collection of genomic information at various molecular resolutions hold promise to accelerate the discovery of new therapeutic targets. A critical step in achieving these goals is to develop efficient clinical prediction models that integrate these diverse sources of high-throughput data. This step is challenging due to the presence of high-dimensionality and complex interactions in the data. For predicting relevant clinical outcomes, we propose a flexible statistical machine learning approach that acknowledges and models the interaction between platform-specific measurements through nonlinear kernel machines and borrows information within and between platforms through a hierarchical Bayesian framework. Our model has parameters with direct interpretations in terms of the effects of platforms and data interactions within and across platforms. The parameter estimation algorithm in our model uses a computationally efficient variational Bayes approach that scales well to large high-throughput datasets.

**Results:**

We apply our methods of integrating gene/mRNA expression and microRNA profiles for predicting patient survival times to The Cancer Genome Atlas (TCGA) based glioblastoma multiforme (GBM) dataset. In terms of prediction accuracy, we show that our non-linear and interaction-based integrative methods perform better than linear alternatives and non-integrative methods that do not account for interactions between the platforms. We also find several prognostic mRNAs and microRNAs that are related to tumor invasion and are known to drive tumor metastasis and severe inflammatory response in GBM. In addition, our analysis reveals several interesting mRNA and microRNA interactions that have known implications in the etiology of GBM.

**Conclusions:**

Our approach gains its flexibility and power by modeling the non-linear interaction structures between and within the platforms. Our framework is a useful tool for biomedical researchers, since clinical prediction using multi-platform genomic information is an important step towards personalized treatment of many cancers. We have a freely available software at: http://odin.mdacc.tmc.edu/~vbaladan.

## 1 Introduction

Recent advances in genome technologies such as microarrays and next-generation sequencing have enabled the measurement of genomic activity at a very detailed resolution (e.g., base pair, single-nucleotide polymorphisms) as well as across multiple molecular levels: the epigenome, transcriptome and proteome. The collection of genomic information at various resolutions holds promise to accelerate the amalgamation of discovery science and clinical medicine [1]. One of the overarching goals of such studies is to relate these genomic data to relevant (patient-specific) clinical outcomes, not only to find significant biomarkers of disease progression/evolution but also to use the biomarkers to develop prediction models for deployment in future therapeutic studies. Furthermore, genomic data are now available from multiple platforms and resolutions for the same individual, thus allowing a researcher to simultaneously query these multiple sources of data to achieve these goals. Such motivating data have been collected under the aegis of The Cancer Genome Atlas (TCGA) project, wherein data from multiple genomic platforms such as gene/mRNA expression, DNA copy number, methylation and microRNA expression profiles are available for multiple tumor types (see http://cancergenome.nih.gov for more details). In addition, the available clinical information, such as stage of disease and survival times, motivates the analytic frameworks that integrate patient-specific data.

One of the main challenges in modeling the statistical dependence between such high-throughput studies is that a large number of measurements (usually in thousands) is available for a relatively small number (usually in tens or hundreds) of patient samples; therefore, classical statistical approaches based on linear models and hierarchical clustering are prone to over-fitting [2,3]. In these situations, [3] recommends accounting for high-dimensionality by using approaches that borrow information across covariates to compensate for the limited information available across samples, which leads to better and more reliable inference. Several approaches have been developed to address these challenges in various contexts. Some examples include linear parametric models and hierarchical clustering for inferring the relation between phenotypes and genomic features [4], hierarchical Bayesian modeling approaches based on linear shrinkage estimators [5], linear canonical correlation analysis [6], intensity-based approaches for merging datasets [7], and regularized linear regression approaches [8].

Although these approaches are computationally efficient, interpretable, and simple, they make two unrealistic assumptions for practical data analysis. First, due to the parametric and linear assumptions, they might miss the underlying non-linear patterns in the data. Second, and more importantly, these non-linear patterns are further amplified in the presence of complex interactions within and between the different platforms that must be modeled while integrating data from these platforms. In this paper, we present a statistical machine learning approach called the hierarchical relevance vector machines (H-RVM) to address these modeling and inferential challenges. Briefly, the framework presented here: (a) models the relation between a relevant clinical outcome (scalar) and high-dimensional covariates/features through a data-adaptive and flexible nonparametric approach,(b) borrows information within and between platforms through a hierarchical Bayesian framework, (c) acknowledges and models the interaction between platforms through nonlinear kernel-based functionals, (d) has parameters that have explicit interpretation as the effects of the platforms and their interactions on the outcome, and (e) uses a computationally efficient variational Bayes approach that can be readily scaled to large datasets.

Our methods are motivated by and applied to a TCGA based glioblastoma multiforme (GBM) dataset, for which we integrate gene (mRNA) and microRNA (miRNA) expression profiles to predict patient survival times^a^. There is an increasing interest in identifying subtypes of GBM based on its gene expression data. The ultimate goal of subtyping GBM is to identify gene expression profiles that are prognostic or predictive of treatment outcomes. The known subtypes of GBM samples in TCGA include pro-neural, neural, classical, and mesenchymal; with the first two classes of which are suspected to differ from the other two in the cell of origin, which is a critical determinant of effective treatment regimens [9]. Differential expressions of miRNAs were recently found to be associated with many diseases, including cancers [10,11]. Previous studies have shown that combining multiple types of data, such as mRNA and miRNA expressions, could significantly improve the accuracy of detecting GBM subtypes, and thereby potentially predict the clinical outcomes [12]. However, methods are lacking to accurately model the effect of interactions between these data types directly on clinical outcomes. Here we show that our non-linear and interaction-based integrative methods have better prediction accuracy than linear alternatives and non-integrative methods that do not account for the interactions between the platforms. We also find several prognostic mRNAs and microRNAs that are related to tumor invasion and that are known to drive tumor metastasis and severe inflammatory response in GBM. In addition, our analysis reveals several interesting mRNA-miRNA interactions that have known implications in the etiology of GBM. The paper is structured as follows. The basic construction of H-RVM is detailed in Section 2. The analysis of GBM data is presented in Section 3, and concluding remarks about the H-RVM framework are presented in Section 4.

## 2 Hierarchical Relevance Vector Machine model

For ease of exposition, we illustrate the model building process of H-RVM using data from two sources: gene/mRNA and miRNA expression measurements. The framework is easily extended to multiple platforms as discussed in Section 4. Suppose, we have data for *N* patients, and **X** and **Y** represent mean-centered and -standardized gene and miRNA expression matrices, with rows corresponding to patients and columns representing the *G* genes and *M* miRNAs, respectively ^b^. Centering and standardizing the gene and miRNA expression matrices remove any systematic mean or scaling effects caused by the use of different data sources, and make them compatible for model fitting. We denote the gene and miRNA expression for the *i*-th patient as row vectors $x_{i}^{T}=(x_{i1},\ldots ,x_{\text{iG}})$ and $y_{i}^{T}=(y_{i1},\ldots ,y_{\text{iM}})$. These covariates are high-dimensional, that is, both *G* and *M* are much larger than *N*; for example, in the GBM data *G*≈12000,*M*≈540,*N*≈250. Based on these measurements, our aim is to predict a relevant clinical outcome, which in our case is the (log-transformed) survival time measured from time of diagnosis to death, denoted by the column vector **t**=(*t*_1_,…,*t*_*N*_) for the *N* patients.

### 2.1 Basic construction

A basic (conceptual) model can be written in a high-dimensional regression setting as,

(1) $$t_{i}=\alpha_{0}+f_{x}(x_{i},\alpha_{1})+f_{y}(y_{i},\alpha_{2})+f_{\left(x⊗y\right)}(x_{i},y_{i},\alpha_{3})+\varepsilon_{i},$$

where *α*_0_ is the overall mean effect and *ε*_*i*_ is the random error; *f*(•)’s, generally referred to as *basis functions*, are chosen to achieve a desired level of flexibility for modeling the effects of **X**,**Y**, and their functions on **t**. Of these functions, *f*_(**x**⊗**y**)_ models the *interactions* between **X** and **Y**, and the remaining basis functions, *f*_**x**_ and *f*_**y**_, respectively, model the main effects of **X** and **Y** for predicting **t**. In most situations the regression coefficients, ***α***=(*α*_0_,***α***_1_,***α***_2_,***α***_3_), linearly relate the covariate effects (i.e., values of the basis functions evaluated at the covariates) to the response. Linear regression is a special case of (1) when all the basis functions are linear, and the response for the *i*-th patient,

(2) $$t_{i}=x_{i}^{T}\alpha_{1}+y_{i}^{T}\alpha_{2}+{\left(x_{i}⊗y_{i}\right)}^{T}\alpha_{3}+\varepsilon_{i},$$

where (**x**_*i*_⊗**y**_*i*_)=(*x*_*i*1_*y*_*i*1_,…,*x*_*i*1_*y*_*iM*_,…,*x*_*iG*_*y*_*i*1_,…,*x*_*iG*_*y*_*iM*_) models the first order interactions between genes and miRNAs and *α*_0_=0 because of the centered covariates. Further, due to the high-dimensional covariates **x**_*i*_’s and **y**_*i*_’s, a penalty is imposed on the regression coefficients ***α***=(***α***_1_,***α***_2_,***α***_3_) to avoid overfitting. The most popular of such penalties is the Lasso because it has many desirable properties for high-dimensional linear regression and variable selection [13,14]. Although (2) with a Lasso penalty is a popular choice for high-dimensional regression, the linearity of the basis functions imposes serious restrictions on the flexibility of the model. For example, (2) does not model nonlinear covariate effects as well as second or higher order interactions between genes and miRNAs.

Through H-RVM, we propose a regression model as a special case of (1), using kernel-based functions to respectively model *f*_**x**_,*f*_**y**_, and *f*_(**x**⊗**y**)_. The kernel functions incorporate nonlinear effects of possible interactions within and between high-dimensional gene and miRNA expression measurements. Further, H-RVM estimates the respective contributions of genes, miRNAs, and their interactions in predicting survival times, which is of primary importance in developing novel drug targets. H-RVM posits the following regression of **t** on **X** and **Y** for the *i*-th patient:

(3) $$\;t_{i}=\beta_{1}f_{x}\{x_{i},\alpha_{1}\}+\beta_{2}f_{y}\{y_{i},\alpha_{2}\}+\beta_{3}f_{\left(x⊗y\right)}\{{\left(x⊗y\right)}_{i},\alpha_{3}\}+\varepsilon_{i},$$

where (**x**⊗**y**)_*i*_=(*x*_*i*1_,…,*x*_*iG*_,*y*_*i*1_,…,*y*_*iM*_) is a vector of length *G*+*M* and ***β***=(*β*_1_,*β*_2_,*β*_3_) is such that its components lie on a probability simplex i.e. *β*_*m*_>0 for *m*=1,2,3 and $\overset{3}{\underset{m=1}{\sum }}\beta_{m}=1$. H-RVM posits different kernels for the data sources and combines them through weights ***β***. The model parameters have the following interpretation: 

•The kernel functions *f*_**x**_(•) and *f*_**y**_(•) model all possible interactions among genes and among miRNAs, respectively, and *f*_(**x**⊗**y**)_(•) models all possible interactions between genes and miRNAs. The three kernels together account for the high-dimensionality and non-linearity of the covariate effects of **X** and **Y** by embedding them in the space of kernels.

•The *m*-th component of ***β***, *β*_*m*_, denotes the influence of the *m*-th source on predicting the log survival time and has the following interpretation: if ***β***={1,0,0}, then (3) corresponds to a functional regression model that predicts **t** (log survival time) with only **X** (gene expressions) as covariates. Conversely, if ***β***={1/3,1/3,1/3}, then (3) corresponds to a regression model, with the platforms and their interactions contributing equally to the prediction of the survival time. In reality, we expect (and show) different contributions from each platform and estimate these weights from the data.

The task now remains to explicitly characterize the functions *f*_**x**_(•), *f*_**y**_(•) and *f*_(**x**⊗**y**)_(•) using multiple kernels, as detailed below.

### 2.2 Multiple kernel learning

Kernel learning (KL) is an approach for nonparametric classification and regression that can be used for predicting **t** based on **X** and **Y**[14]. First, for simplicity, assume that we want to predict **t** based on **X**. KL posits the following relation between **t** and **X**

(4) $$t_{i}=\alpha_{0}+\overset{N}{\underset{j=1}{\sum }}\alpha_{j}K(x_{j},x_{i}|\sigma^{2})+\varepsilon_{i}\Rightarrow t=K^{T}\alpha +\varepsilon ,$$

where *σ*^2^ is a kernel-specific “bandwidth” parameter and depends on the choice of kernel, *K*(•) (detailed later in the section) and ***ε***=(*ε*_1_,…,*ε*_*N*_) is the (white-noise) error. The primary parameter of interest is ***α***=(*α*_0_,…,*α*_*N*_)^*T*^, and *α*_1_,…,*α*_*N*_ correspond to weights assigned to the features for *N***x**_*j*_’s. Support Vector Machine (SVM) and Relevance Vector Machine (RVM) are canonical examples of KL [14]. We prefer RVM because of its probabilistic interpretation and other optimal properties compared to those of SVM [15]. There are cases, however, where one feature matrix may not fit the data well. Based on this observation, Multiple Kernel Learning (MKL) extends (4) and replaces **K** by a weighted average of *L* feature matrices ${\left\{K_{l}\right\}}_{l=1}^{L}$,

(5) $$\begin{array}{ll} t_{i} & =\alpha_{0}+\overset{N}{\underset{j=1}{\sum }}\alpha_{j}\overset{L}{\underset{l=1}{\sum }}\beta_{l}K_{l}(x_{j},x_{i}|\sigma_{l}^{2})+\varepsilon_{i}\;\Rightarrow \;\\ t & =\beta_{1}K_{1}^{T}\alpha +\ldots +\beta_{l}K_{l}^{T}\alpha +\ldots +\beta_{L}K_{L}^{T}\alpha +\varepsilon . \end{array}$$

MKL improves the flexibility of KL by introducing *L* bandwidth parameters ${\left\{\sigma_{l}^{2}\right\}}_{l=1}^{L}$ and *L* weights for feature matrices ***β***=(*β*_1_,…,*β*_*L*_)^*T*^. A variety of approaches exist to learn ${\left\{\sigma_{l}^{2}\right\}}_{l=1}^{L}$, ***β***, and ***α*** for MKL (for details see [14,16,17]). Note that in all these works the data source (i.e., **X**) remains the same for both KL and MKL. The H-RVM framework developed in this article extends KL to include multiple data sources and their interactions, and uses a learning algorithm similar to the MKL framework.

Because the three data sources (gene expressions, miRNA expressions, and their interactions) can be used separately for predicting the log survival time, it is reasonable to combine their predictions to obtain more reliable estimates. To this end, H-RVM combines respective predictions obtained from different sources obtained using KL (4) through a weighted average, and chooses appropriate weights using MKL (5). Similar to (4), $K_{1}^{T}\alpha ,K_{2}^{T}\alpha$, and $K_{3}^{T}\alpha$ are the predicted values of **t** that correspond to genes, miRNAs, and their interactions, respectively. Using (5), we combine the predictions ${\left\{K_{i}^{T}\alpha \right\}}_{i=1}^{3}$ through the weight vector ***β***=(*β*_1_,*β*_2_,*β*_3_) to model **t** as

(6) $$t=(\beta_{1}K_{1}^{T}+\beta_{2}K_{2}^{T}+\beta_{3}K_{3}^{T})\alpha +\varepsilon =K_{\beta }^{T}\alpha +\varepsilon .$$

We further constrain ***β*** such that its components lie on a probability simplex, i.e., $\overset{3}{\underset{m=1}{\sum }}\beta_{m}=1$. This constraint ensures that the joint (convolved) kernel, **K**_***β***_, is positive definite and that *β*_*m*_ denotes the influence of the *m*-th source in predicting the log survival time. Note that H-RVM is a special case of (3) with $f_{x}(x_{i},\alpha )\equiv k_{1,i}^{T}\alpha$, $f_{y}(y_{i},\alpha )\equiv k_{2,i}^{T}\alpha$, and $f_{\left(x⊗y\right)}({\left(x⊗y\right)}_{i},\alpha )\equiv k_{3,i}^{T}\alpha$, where **k**_*m*,*i*_ is the *i*-th column of **K**_*m*_. Given ${\left\{K_{i}\right\}}_{i=1}^{3}$, MKL can be used to learn ***α*** and ***β***.

Although similar to (5), (6) differs in two important ways. First, (6) obtains kernels using (4) for different data sources, namely gene expression, miRNA expression, and their interaction. Second, we allow for dependence between data sources via the interaction kernel (**K**_3_), but MKL does not; instead MKL uses a convex combination of the different kernels from the same data source to aid prediction.

The learning algorithm of H-RVM is independent of the choice of kernels, but in this work we use a Gaussian radial basis function (RBF) kernel (denoted by **K**) [14]. The RBF kernel maps the *m*-th high-dimensional covariate to its feature space that is represented as feature matrix **K**_*m*_. The feature matrices **K**_1_,**K**_2_, and **K**_3_ correspond to genes, miRNAs, and interactions, and their (*i*,*j*)-th entries are as follows:

(7) $$\begin{array}{ll} {\left(K_{1}\right)}_{\text{ij}} & =e^{-\frac{∥x_{i}-x_{j}{∥}^{2}}{2\sigma_{1}^{2}}}=K(x_{i},x_{j}|\sigma_{1}^{2}),\;\\ {\left(K_{2}\right)}_{\text{ij}} & =e^{-\frac{∥y_{i}-y_{j}{∥}^{2}}{2\sigma_{2}^{2}}}=K(y_{i},y_{j}|\sigma_{2}^{2}),\\ {\left(K_{3}\right)}_{\text{ij}} & =e^{-\frac{∥{\left(x⊗y\right)}_{i}-{\left(x⊗y\right)}_{j}{∥}^{2}}{2\sigma_{3}^{2}}}=K({\left(x⊗y\right)}_{i},{\left(x⊗y\right)}_{j}|\sigma_{3}^{2}), \end{array}$$

where $\sigma_{m}^{2}$ is the “bandwidth” parameter of the *m*-th kernel matrix and is chosen *a priori* through cross-validation (see [14] for details). The other choices of kernels include polynomial kernels and matern kernels [18]. To account for the overall mean (or intercept) in (1), an extra row of 1’s is appended to the feature matrices in (7); therefore, ${\left\{K_{i}\right\}}_{i=1}^{3}$ hereafter have dimensions (*N*+1)×*N*.

### 2.3 Generative Bayesian model for H-RVM

H-RVM reformulates (6) as a hierarchical Bayesian model for greater flexibility and better interpretation of its parameters. This reformulation serves two important purposes. First, H-RVM is interpreted as a hierarchical Bayesian extension of RVM [15], which is a special case of Bayesian KL. Second, instead of using MKL methods, H-RVM learns parameters ***α*** and ***β*** from **t**,**X**, and **Y** using the variational learning algorithm of hierarchical kernel learning (HKL) [14,16].

H-RVM posits the following generative model for the (noisy) log survival time measurements **t**. Similar to MKL, $K_{\beta }^{T}\alpha$ represents the mean of **t**. The error distribution is Gaussian with mean 0 and precision parameter *γ* (8). Further, we impose a Gamma prior on *γ* such that

(8) $$\begin{array}{ll} t|\alpha ,\beta ,\gamma ,X,Y∼N(t|K_{\beta }^{T}\alpha ,\gamma^{-1}I), & \; \end{array}$$

(9) $$\begin{array}{ll} \gamma ∼\text{Gamma}(\gamma |c_{\gamma },d_{\gamma }), & \; \end{array}$$

where N(.|μ,Σ) represents a multivariate Gaussian distribution with mean ***μ*** and covariance matrix ***Σ*** and Gamma(.|*c*_•_,*d*_•_) represents a Gamma distribution with respective shape and rate parameters *c*_•_ and *d*_•_.

Motivated by the “automatic relevance determination” idea of RVM, we impose independent Gaussian priors on the *α*_*j*_’s with the same mean 0 and different precision parameters *ϕ*_*j*_’s (10), where *ϕ*_*j*_ controls (*a priori*) predictive power of the *j*-th feature vector from the three data sources for the log survival time. A large *ϕ*_*j*_ indicates low predictive power. We also impose independent Gamma priors on the *ϕ*_*j*_’s,

(10) $$\begin{array}{ll} \alpha |\phi ∼N(\alpha |0,\text{diag}(\phi^{-1})), & \; \end{array}$$

(11) $$\begin{array}{ll} \phi ∼\overset{N}{\underset{j=0}{\prod }}\text{Gamma}(\phi_{j}|c_{\phi },d_{\phi }), & \; \end{array}$$

where $\phi =(\phi_{0},\phi_{{}_{1}},\ldots ,\phi_{n})$. This setting forces many *α*_*j*_’s *a posteriori* to be near 0 with high precision. Most of the variance in **t** is explained by a small number of feature vectors that correspond to nonzero *α*_*j*_’s. These feature vectors are the “relevance vectors” of H-RVM that have the following three characteristics: they prevent over-fitting, represent a parsimonious description of the data, and correspond to feature vectors that are most predictive of the log survival time. An equivalent prior setting is found by marginalizing the *ϕ*_*j*_’s from the joint distribution of ***α*** and ***ϕ*** above, which imposes a multivariate Student’s *t* prior on ***α*** with mean **0**.

Finally, we impose a Dirichlet prior on ***β*** to ensure that its components lie on a probability simplex:

(12) $$\begin{array}{ll} \beta =(\beta_{1},\beta_{2},\beta_{3})∼\text{Dirichlet}(\beta |a_{1},a_{2},a_{3}), & \; \end{array}$$

where the *m*-th component of ***β***, *β*_*m*_, denotes the influence of *m*-th source in predicting the log survival time.

The hierarchical Bayesian model (8) – (12) specifies a complete sampling model for the H-RVM framework. It can also be interpreted as a probabilistic approach for combining the predictions of log survival times from the three RVMs respectively corresponding to gene expressions, miRNA expressions, and their interactions. H-RVM introduces an additional hierarchy and combines the predictions of these three RVMs as a weighted average, with the weights generated from a Dirichlet distribution (12). The increased flexibility of H-RVM over RVM comes at the cost of analytic intractability of the posterior distributions of the H-RVM parameters. Estimation of the posterior distributions of the H-RVM’s parameters can proceed via either simulation-based Markov chain Monte Carlo (MCMC) approaches or deterministic variational Bayes approaches. Given the complexity and size of high-throughput data in general and GBM data in particular, MCMC approaches tend to be computationally expensive and slow. We employ variational Bayes methods from HKL [16] and obtain the analytically tractable variational posterior distribution, *q*(***α***,***β***,***ϕ***,*γ*|**t**,**X**,**Y**,*c*_*ϕ*_,*d*_*ϕ*_,*c*_*γ*_,*d*_*γ*_,*a*_1_,*a*_2_,*a*_3_), that approximates analytically intractable true posterior distribution, *p*(***α***,***β***,***ϕ***,*γ*|**t**,**X**,**Y**,*c*_*ϕ*_,*d*_*ϕ*_,*c*_*γ*_,*d*_*γ*_,*a*_1_,*a*_2_,*a*_3_). This approximation achieves analytic tractability by assuming that ***α***,***β***,***ϕ***, and *γ* are independent under the variational posterior distribution. The analytic tractability leads to improved computational efficiency of the variational Bayes approach over sampling-based MCMC approaches. The derivations for variational posterior distributions are provided in Appendix A Appendix: Variational inference for H-RVM.

## 3 Data analysis

We apply the H-RVM approach to the GBM data as introduced in Section 1. GBM was one of the first cancers evaluated by the TCGA. GBM data have multiple molecular measurements on over 500 samples that include gene expression, copy number, methylation and microRNA expression. TCGA datasets are available at http://tcga-data.nci.nih.gov/tcga/. The dataset we analyze here includes information about the gene expressions (11972 probes), miRNA expressions (534 probes), and (uncensored) survival times for matched patient samples (248).

To remove the irrelevant noise variables before model fitting, we prescreened the gene and miRNA probes as follows. We performed univariate regression of the log survival times on the gene expression values, obtained p-values, and retained gene and miRNA probes that cross a liberal p-value threshold (≤ 0.05 here) – to balance the practical and statistical significance. This pre-selection identifies 1747 and 43 gene expression and miRNA probes, respectively, for downstream modeling. All our analyses and comparisons were based on these selected gene and miRNA probes.

We compare the predictions of H-RVM and three linear methods: penalized regressions (2) with the Lasso penalty [13] and with the following covariates: *i.* gene expressions (Gene-Lasso), *ii.* miRNA expressions (MiRNA-Lasso), and *iii.* both gene and miRNA expressions, and their first order interactions (Interaction-Lasso). We randomly split the GBM survival data into a training data and a test data with 223 (90%) and 25 (10%) patients, respectively. H-RVM, Gene-Lasso, MiRNA-Lasso, and Interaction Lasso are fit using the gene and miRNA expressions and log survival times in the training data. The variational inference algorithm is used for fitting H-RVM (see Appendix A Appendix: Variational inference for H-RVM). The R package glmnet is used for the three penalized linear regressions [19,20]. The log survival times of the test data are predicted for the four methods using the model fits on the training data. The mean squared prediction errors (MSPEs) are respectively calculated for the four models as the average of the squared difference between the observed and predicted values for the test data. This process of randomly splitting the GBM survival data into training+test data and fitting the four models is repeated 50 times. The results are summarized below.

**Figure 1:**
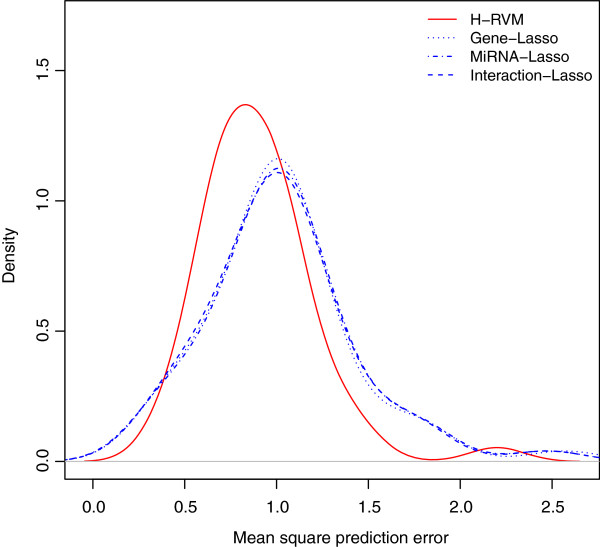
**Kernel density estimates.** Kernel density estimates (KDEs) of mean square prediction errors (MSPEs) for H-RVM, Gene-Lasso, MiRNA-Lasso, and Interaction-Lasso. The GBM survival data is randomly split 50 times into training and test data, all four models are fit on the training data, and MSPEs for log survival times are calculated for the test data using the model fit on training data. The x-axis represents the MSPEs and the y-axis represents the respective KDEs for H-RVM (in solid red), Gene-Lasso (in dotted blue), MiRNA-Lasso (in dotted and dashed blue), and Interaction-Lasso (in dashed blue).

Figure 1 shows the prediction results for H-RVM, Gene-Lasso, MiRNA-Lasso, and Interaction-Lasso using the kernel density estimates (KDEs) of the MSPEs for the 50 random splits. The KDEs of the MSPEs for H-RVM is shifted to lower values than those for the three penalized linear regressions. The KDEs of the MSPEs for Gene-Lasso, MiRNA-Lasso, and Interaction-Lasso are close to each other, which implies that the MSPEs for these models are fairly similar. Two observations arise from these results. First, the results indicate that penalized linear regression with the Lasso penalty does not lead to improved performance after accounting for interactions among covariates, which has been well-established in literature [19]. Second, the prediction results of the penalized linear regressions do not improve after modeling the first order interactions among genes and miRNAs, thus indicating the presence of non-linear genomic effects and second or higher order interactions among them. For this case study, we see that H-RVM performs better than the penalized linear regression methods. This may be because of H-RVM accounts for the nonlinear effects of genes and miRNAs and models the interactions within genes, within miRNAs, and between genes and miRNAs. Further, because we model the log survival time, the gain for survival time predictions is, in fact, *exponentially higher* for H-RVM compared to those for the other methods.

**Figure 2:**
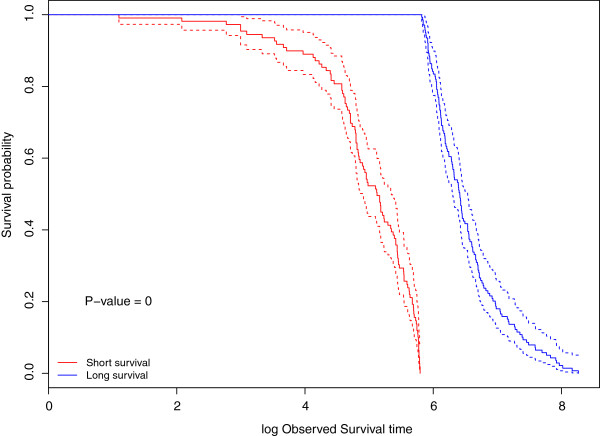
**Survival probability curves for TCGA data.** Survival probability curves for log survival time in the TCGA GBM data. The solid lines are the Kaplan-Meier estimates of survival probabilities for the patients predicted to have long survival times (in blue) and for the patients predicted to have short survival times (in red), respectively. The patients are assigned to the long and short survival groups based on the estimates of log survival times obtained from H-RVM. The dotted lines indicate point-wise 95% confidence intervals for the survival probabilities. The p-value of the log rank test is 0.

**Figure 3:**
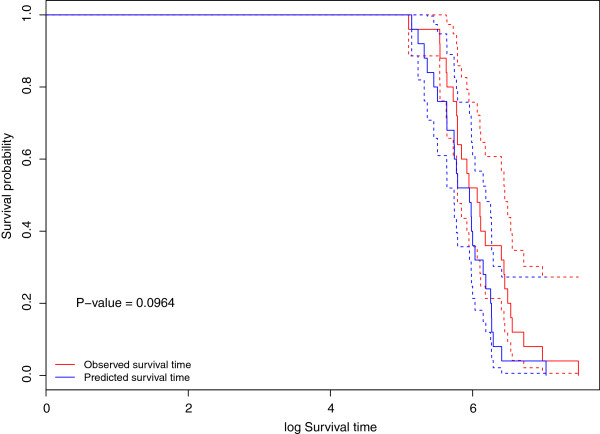
**True and predicted survival probability curves.** Survival probability curves for the observed log survival time and predicted log survival time (using H-RVM) of the patients in the test data with minimum mean square prediction error. The solid lines are the Kaplan-Meier estimates of survival probabilities for the predicted (in blue) and observed (in red) log survival times in the test data. The dotted lines indicate point-wise 95% confidence intervals for the survival probabilities. The p-value of the log rank test is 0.0964.

We compared the performance of the predictions of the log survival times from H-RVM and the observed survival times using Kaplan-Meier estimates of the survival curves. We used the R package survival to perform the log rank test and estimate the Kaplan-Meier survival curves [21]. Figure 2 compares the survival probability curves of the log survival times of patients predicted to be in the long and short survival groups by H-RVM. The patients are assigned to the long and short survival groups based on a median cut-off of the predicted log survival times obtained from H-RVM. The p-value of the log rank test that the two survival curves are the same is close to 0, indicating that the survival group predictions of H-RVM closely agree with the observed survival groups of the patients. In addition, Figure 3 compares the actual survival probability curves of the observed and predicted log survival times of patients in the test data with the minimum MSPE. The p-values and the survival probability curves indicate that the log survival time predictions of H-RVM agree closely agree with the observed log survival times, as well.

**Figure 4:**
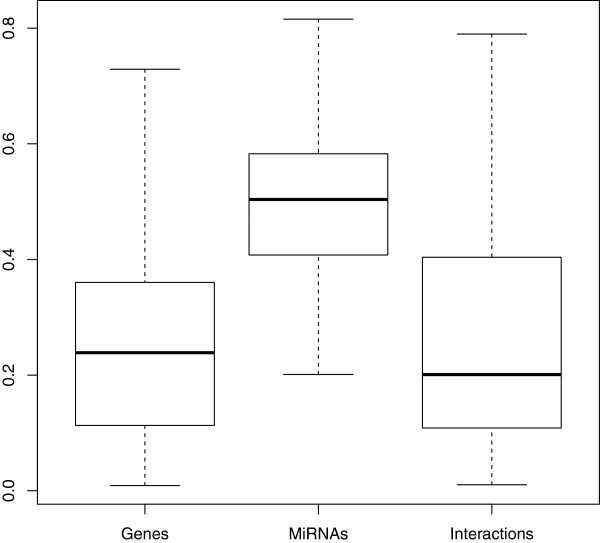
**Boxplots of weights.** Boxplots of weights ***β***=(*β*_1_,*β*_2_,*β*_3_) of gene expressions, miRNA expressions, and their interactions in predicting log survival time. The GBM survival data is randomly split 50 times into training and test data, H-RVM is fit on the training data, and ***β*** is obtained from the fit on training data. The y-axis shows the distributions of respective weights for gene expressions, miRNA expressions, and their interactions in predicting log survival time of patients across 50 random splits of the GBM survival data.

One of the additional gains of our modeling framework is the determination of which platform has a more profound influence on predicting the response, as captured by the weight parameter ***β***. Figure 4 shows the estimates of the weights ***β*** for predicting the log survival time of the patients for gene expression, miRNA expressions, and their interactions obtained from H-RVM. The medians of the weights (25% and 75% quartiles) for the three data sources are 0.239 (0.113 and 0.360), 0.504 (0.408 and 0.583), and 0.201 (0.108 and 0.404), respectively. Interestingly, H-RVM shows that miRNAs have better predictive power than genes in predicting the log survival times of patients in the GBM data. The nonzero weight for interactions between gene and miRNA expressions further confirms the presence of nonlinear interactions.

To gain biological insights into our results, we performed a functional analysis of our model fitting results. We used Ingenuity Pathway Analysis software to perform functional analysis on selected significant genes used in fitting H-RVM. We used targetHub [22] to find the known and predicted interactions between significant genes and miRNAs. mirTarBase, a curated database of experimentally validated miRNA targets, was our choice as a source of known gene and miRNA interactions [23]. To identify the predicted gene and miRNA interactions, we used targetScan data [24] to filter out miRNA-gene interactions in which the miRNA and gene effects on survival were concordant, since discordant behavior is expected in biological systems for a direct interaction between miRNA and its targets.

**Figure 5:**
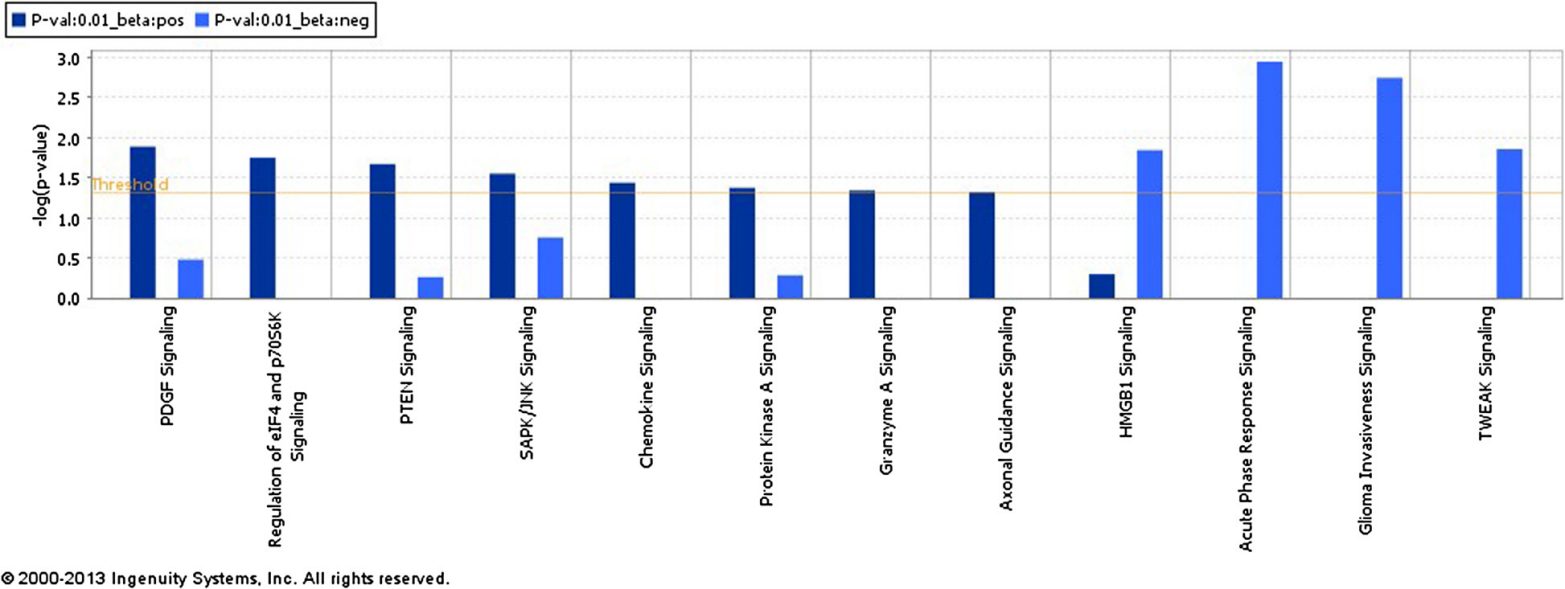
**Comparison of the signaling pathways.** Comparison of the signaling pathways associated with significant prognostic genes in Glioblastoma multiforme.

Pathway analyses indicates that the anti-survival genes (i.e., genes with negative effects on survival times) are enriched with signaling pathways related to tumor invasion (see Figure 5). HMGB1 and TWEAK signaling pathways, which are known to drive tumor metastasis and severe inflammatory responses in GBM and other cancers, are associated with these genes [25-28]. Pro-survival genes are represented by PDGF, PTEN and other signaling pathways. It is well-established that the PDGF signaling pathway dominates the proneural subgroup, which correlates with a good survival time for patients with GBM [29]. The functional terms cellular movement and cell-to-cell signaling and interaction pathways are enriched for anti-survival genes, reinforcing their role in invasive GBM.

The target analysis of miRNA revealed 22 known interactions between 8 miRNAs and 20 genes, as shown in Table 1. Four of these eight miRNAs (hsa-miR-31, hsa-miR-146b, hsa-miR-221 and hsa-miR-222) were previously identified as anti-survival markers of GBM [30]. Mir-21 is an established marker of GBM and is known to target many tumor suppressor genes [31]. Mir-34a expression is higher in other GBM subtypes compared to that in the pro-survival proneural glioma subtype [32]. The anti-survival patterns of all these miRNAs indicate that these gene and miRNA interactions can be targeted for therapy of GBM subgroups with expected poor survival. We also identified 1006 predicted interactions (by TargetScan) between 31 miRNA and 484 genes that are significant (see Additional file 1).

**Table 1:** List of known gene-microRNA interactions identified as significant in the H-RVM model using target analysis

**Gene symbols**	**microRNA**
FOXP3, YY1, KLF13, ETS1	hsa-mir-31
FOXO3, DDIT4	hsa-mir-221
ATAT1	hsa-mir-23a
FOXO3	hsa-mir-222
FGG, CPEB3, FGB, PIK3R1	hsa-mir-29a
PDGFB	hsa-mir-146b
PDCD4, TOPORS, BASP1, MARCKS, TP53BP2	hsa-mir-21
SIRT1, YY1, E2F3, CDC25C	hsa-mir-34a

## 4 Conclusions and future work

We have presented an integrative framework, H-RVM, that generalizes the multiple kernel learning framework for integrating high-dimensional data from multiple sources, incorporating within and between platform interactions to develop a prediction model for clinical outcomes. We applied H-RVM to a high-dimensional TCGA GBM data to predict patient survival times using two data sources: gene and miRNA expressions, and found that the predictive performance of H-RVM is better than those of linear methods that do not model nonlinear effects and interactions. We hypothesize that H-RVM gains flexibility and power by modeling the non-linear interaction structures between gene and miRNA expressions. H-RVM will be a useful tool for biomedical researchers, as clinical prediction using multi-platform genomic information is an important step towards identifying personalized treatments for many cancers. We have code for fitting H-RVM that is freely available at the corresponding author’s website (see Additional file 2).

Although we have presented the application of H-RVM in the context of two platforms, the framework is general and can be extended and adapted to data from multiple platforms with different distributional assumptions. This will essentially entail a generalization of the H-RVM model by assuming additional terms for the different platforms. One key issue that warrants further investigation is an increase in the number of (multiplicative) between-platform interaction terms. We used the computationally efficient variational Bayes methods, which are extremely useful for handling large datasets from projects such as TCGA. In addition, [17] presents more scalable versions of HKL and MKL that can be adapted to our framework. Our future work will concentrate on extending the H-RVM framework using Bayesian spike and slab priors to select variables from the interacting covariates before embedding the data in the space of kernels, as well as incorporating uncertainty estimations of the scale parameters – thus aiding the joint model building process.

## Endnotes

^a^ We use gene and mRNA interchangeably to mean transcript-level expression.

^b^ We use bold lowercase and uppercase alphabets to denote column vectors and matrices, respectively.

## A Appendix: Variational inference for H-RVM

Following the hierarchic kernel learning algorithm (HKL) of [16], we provide the derivation for the variational inference algorithm that estimates the variational posterior distributions for the parameters of H-RVM. Our interest lies in the posterior distributions of ***α*** and ***β*** that are obtained using the Bayesian model (8) – (12). Unlike RVM, the posterior distributions of ***α*** and ***β*** in H-RVM are analytically intractable. There are several techniques that can be used to obtain these posteriors distributions. We employ the variational Bayes methods from HKL [16] and obtain analytically tractable variational posterior distribution, *q*(***α***,***β***,***ϕ***,*γ* |**t**,**X**,**Y**,*c*_*ϕ*_,*d*_*ϕ*_,*c*_*γ*_,*d*_*γ*_,*a*_1_,*a*_2_,*a*_3_), that approximates analytically intractable true posterior distribution, *p*(***α***,***β***,***ϕ***,*γ*|**t**,**X**,**Y**,*c*_*ϕ*_,*d*_*ϕ*_,*c*_*γ*_,*d*_*γ*_,*a*_1_,*a*_2_,*a*_3_). The variational approach minimizes the Kullback-Liebler (KL) divergence between *q*(***α***, ***β***, ***ϕ***,*γ*|**t**, **X**,**Y**,*c*_*ϕ*_,*d*_*ϕ*_,*c*_*γ*_, *d*_*γ*_,*a*_1_,*a*_2_,*a*_3_) and *p*(***α***,***β***, ***ϕ***,*γ*|**t**,**X**,**Y**, *c*_*ϕ*_,*d*_*ϕ*_,*c*_*γ*_,*d*_*γ*_,*a*_1_,*a*_2_,*a*_3_). This approximation achieves analytic tractability by assuming that ***α***,***β***, ***ϕ***, and *γ* are independent under *q*(***α***,***β***,***ϕ***, *γ*|**t**,**X**,**Y**,*c*_*ϕ*_,*d*_*ϕ*_,*c*_*γ*_, *d*_*γ*_,*a*_1_,*a*_2_,*a*_3_). Therefore,

(13) $$\begin{array}{l} q(\alpha ,\beta ,\phi ,\gamma |t,X,Y,c_{\phi },d_{\phi },c_{\gamma },d_{\gamma },a_{1},a_{2},a_{3})\\ \;=q(\alpha )q(\beta )q(\phi )q(\gamma ), \end{array}$$

where we have suppressed the conditioning on the data and hyperparameters for the variational posteriors on the right. Notice that the factorization (13) alone guarantees the analytic tractability of *q*(***α***,***β***,***ϕ***,*γ*|**t**,**X**,**Y**,*c*_*ϕ*_,*d*_*ϕ*_,*c*_*γ*_,*d*_*γ*_,*a*_1_,*a*_2_,*a*_3_), and we do not assume any distributional form for the *q*’s. Following [16] and [14], the variational posterior distributions are derived as

(14) $$\begin{array}{lll} \log q(\alpha ) & \propto E_{\beta ,\phi ,\gamma }\left[p(\alpha ,\beta ,\phi ,\gamma ,t,X,Y|\;\right) & \;\\ & \left(\;\;\;\;\;c_{\phi },d_{\phi },c_{\gamma },d_{\gamma },a_{1},a_{2},a_{3})\right],\; \end{array}$$

(15) $$\begin{array}{lll} \log q(\beta ) & \propto E_{\alpha ,\phi ,\gamma }\left[p(\alpha ,\beta ,\phi ,\gamma ,t,X,Y|\;\right) & \;\\ & \left(\;\;\;\;\;c_{\phi },d_{\phi },c_{\gamma },d_{\gamma },a_{1},a_{2},a_{3})\right],\; \end{array}$$

(16) $$\begin{array}{lll} \log q(\phi ) & \propto E_{\alpha ,\beta ,\gamma }\left[p(\alpha ,\beta ,\phi ,\gamma ,t,X,Y|\;\right) & \;\\ & \left(\;\;\;\;\;c_{\phi },d_{\phi },c_{\gamma },d_{\gamma },a_{1},a_{2},a_{3})\right],\; \end{array}$$

(17) $$\begin{array}{lll} \log q(\gamma ) & \propto E_{\alpha ,\beta ,\phi }\left[p(\alpha ,\beta ,\phi ,\gamma ,t,X,Y|\;\right) & \;\\ & \left(\;\;\;\;\;c_{\phi },d_{\phi },c_{\gamma },d_{\gamma },a_{1},a_{2},a_{3})\right],\; \end{array}$$

where all expectations are with respect to the variational posterior distributions. Hereafter, we will denote $E_{•}[f]$ as 〈*f*〉_•_ for notational simplicity.

Following [16] and using (14), the variational posterior distribution of ***α***,

(18) $$\begin{array}{ll} q(\alpha ) & =N(\mu_{\alpha },\Sigma_{\alpha });\;\\ \Sigma_{\alpha } & ={\left[{\langle \gamma \rangle }_{\gamma }{\langle K_{\beta }K_{\beta }^{T}\rangle }_{\beta }+\text{diag}({\langle \phi \rangle }_{\phi })\right]}^{-1},\;\\ \mu_{\alpha } & ={\langle \gamma \rangle }_{\gamma }\Sigma_{\alpha }{\langle K_{\beta }\rangle }_{\beta }t. \end{array}$$

Following [16] and using (16), the variational posterior distribution of ***ϕ***,

(19) $$\begin{array}{lll} \;q(\phi ) & =\overset{N}{\underset{j=0}{\prod }}q(\phi_{j}),\text{where}\; & \;\\ \;q(\phi_{j}) & =\text{Gamma}\left(\right)\frac{1}{2}\;+\;c_{\phi },\frac{1}{2}{\langle \alpha_{i}^{2}\rangle }_{\alpha }\;+\;d_{\phi })),\text{for}\;j=1,\ldots ,\mathrm{N.}\; \end{array}$$

Following [16] and using (17), the variational posterior distribution of *γ*,

(20) $$\begin{array}{ll} q(\gamma ) & =\text{Gamma}\left(\right)\frac{N}{2}+c_{\gamma },\frac{1}{2}{\langle ∥e{∥}^{2}\rangle }_{\alpha ,\beta }+d_{\gamma })),\text{where}\\ ∥e{∥}^{2} & =∥t-K_{\beta }^{T}\alpha {∥}^{2}. \end{array}$$

All the expectations above are determined using 〈***α***〉_***α***_,〈***αα***^*T*^〉_***α***_,〈***ϕ***〉_***ϕ***_, and 〈*γ*〉_*γ*_, which are available from the distributional forms of *q*(***α***),*q*(***ϕ***), and *q*(*γ*) in (18) – (20). Specifically,

$$\begin{array}{rll} {\langle \alpha \rangle }_{\alpha } & =\mu_{\alpha },\;{\langle \alpha \alpha^{T}\rangle }_{\alpha }=\Sigma_{\alpha }+\mu_{\alpha }\mu_{\alpha }^{T},\; & \;\\ {\langle \phi_{j}\rangle }_{\phi_{j}} & =\frac{\frac{1}{2}+c_{\phi }}{\frac{1}{2}({\left(\Sigma_{\alpha }\right)}_{\text{jj}}+{\left(\mu_{\alpha }\mu_{\alpha }^{T}\right)}_{\text{jj}})+d_{\phi }},\; & \;\\ {\langle \gamma \rangle }_{\gamma } & =\frac{\frac{N}{2}+c_{\gamma }}{\frac{1}{2}{\langle ∥e{∥}^{2}\rangle }_{\alpha ,\beta }+d_{\gamma }},\; & \;\\ {\langle ∥e{∥}^{2}\rangle }_{\alpha ,\beta } & =\overset{N}{\underset{n=1}{\sum }}t_{n}^{2}-2\overset{N}{\underset{n=1}{\sum }}t_{n}\overset{3}{\underset{m=1}{\sum }}{\langle \beta_{m}\rangle }_{\beta }{\langle \alpha^{T}\rangle }_{\alpha }k_{\text{mn}}\; & \;\\ & \;+\overset{3}{\underset{m=1}{\sum }}\overset{3}{\underset{l=1}{\sum }}{\langle \beta_{m}\beta_{l}\rangle }_{\beta }\Omega_{\text{ml}},\; & \;\\ \Omega_{\text{ml}} & =\overset{N}{\underset{n=1}{\sum }}k_{\text{mn}}^{T}{\langle \alpha \alpha^{T}\rangle }_{\alpha }k_{\text{ln}}.\; & \; \end{array}$$

Instead of *q*(***β***), its non-normalized version *q*^∗^(***β***) is available from [16] as

(21) $$\;\begin{array}{rl} q^{*}(\beta ) & =\overset{3}{\underset{m=1}{\prod }}\beta_{m}^{a_{m}-1}\exp\left(\right)\;\;-\;\frac{{\langle \gamma \rangle }_{\gamma }}{2}\left(\right)\beta^{T}\Omega \beta \;-\;2\beta^{T}b))\;)),\text{where}\\ \Omega_{\text{ml}} & =\overset{N}{\underset{n=1}{\sum }}k_{\text{mn}}^{T}{\langle \alpha \alpha^{T}\rangle }_{\alpha }k_{\text{ln}},\text{for}m,l=1,2,3\;\text{and}\\ b_{m} & ={\langle \alpha^{T}\rangle }_{\alpha }K_{m}t,\;\text{for}m=1,2,3. \end{array}$$

Following [16], calculate 〈***β***〉_***β***_,〈log***β***〉_***β***_, and 〈***ββ***^*T*^〉_***β***_, as follows. Sample *S****β***’s from Dirichlet(*a*_1_,*a*_2_,*a*_3_) and estimate the expectations as ${\langle f(\beta )\rangle }_{\beta }\approx \overset{S}{\underset{s=1}{\sum }}f(\beta_{s})w(\beta_{s})$ where *f*(***β***)≡***β***, log***β***,and***ββ***^*T*^, respectively, and

() $$w(\beta_{s})=\frac{\exp\left(\right)-\frac{{\langle \gamma \rangle }_{\gamma }}{2}\left(\right)\overset{T}{\underset{s}{\beta }}\Omega \beta_{s}-2\beta_{s}^{T}b))))}{\overset{S}{\underset{i=1}{\sum }}\exp\left(\right)-\frac{{\langle \gamma \rangle }_{\gamma }}{2}\left(\right)\overset{T}{\underset{i}{\beta }}\Omega \beta_{i}-2\beta_{i}^{T}b))))}.$$

The analytic tractability of *q*(***α***,***β***,***ϕ***,*γ*|**t**,**X**,**Y**,*c*_*ϕ*_,*d*_*ϕ*_,*c*_*γ*_, *d*_*γ*_,*a*_1_,*a*_2_,*a*_3_) in variational inference guarantees that the marginal variational distribution (or likelihood) of the data *q*(**t**,**X**,**Y**|*c*_*ϕ*_,*d*_*ϕ*_,*c*_*γ*_,*d*_*γ*_,*a*_1_,*a*_2_,*a*_3_) is also analytically tractable. Estimate hyperparameters *c*_*ϕ*_,*d*_*ϕ*_,*c*_*γ*_,*d*_*γ*_,*a*_1_,*a*_2_, and *a*_3_ as

$$\underset{c_{\phi },d_{\phi },c_{\gamma },d_{\gamma },a_{1},a_{2},a_{3}}{\text{arg max}}\log q(t,X,Y|c_{\phi },d_{\phi },c_{\gamma },d_{\gamma },a_{1},a_{2},a_{3}),$$

which is the type II maximum likelihood procedure as recommended in [16]. The kernel parameters ${\left\{\sigma_{i}^{2}\right\}}_{i=1}^{3}$ are learned respectively from three RVMs for each of the three sources using cross-validation as recommended by [15].

## Competing interests

The authors declare that they have no competing interests.

## Authors’ contributions

VB conceived the research. SS and VB worked out the detailed algorithms and derivations. SS implemented the software and conducted the data analysis. WW and VB oversaw the entire research. WW and GM provided the biological insights into the findings. CO provided insights into the predictive analysis. All authors contributed towards writing the manuscript.

## Supplementary Material

Additional file 1 **mRNA-miRNA-predicted-interactions.xlsx.** Excel file containing all predicted mRNA and microRNA interactions flagged as significant in our analysis. The file is available at: http://odin.mdacc.tmc.edu/~vbaladan/Veera_Home_Page/Software_files/mRNA-miRNA-predicted-interactions.xlsx. Click here for file

Additional file 2 **hrvm-0.1.1.tar.gz.** R package for fitting H-RVM available at: http://odin.mdacc.tmc.edu/~vbaladan/Veera_Home_Page/Software_files/hrvm_0.1.1.tar.gz. Click here for file
