# Supplementary material for: Integrating multi-platform genomic data using hierarchical Bayesian relevance vector machines
Source: EURASIP J Bioinform Syst Biol. 2013 Jun 28;2013(1):9. doi: 10.1186/1687-4153-2013-9 (PMC3726335; doi:10.1186/1687-4153-2013-9)
Supplement: Additional file 2 — hrvm-0.1.1.tar.gz. R package for fitting H-RVM available at: http://odin.mdacc.tmc.edu/~vbaladan/Veera_Home_Page/Software_files/hrvm_0.1.1.tar.gz. [file 1687-4153-2013-9-S2.gz › hrvm/doc/hrvm-manual.pdf]

Package ‘hrvm’

August 14, 2012

Type Package

Title Hierarchical Relevance Vector Machines (H-RVM) For Integrating Multiplatform Genomic Data

Version 0.1.1

Date 2012-08-16

Author Sanvesh Srivastava <sanvesh@gmail.com>

Maintainer Sanvesh Srivastava <sanvesh@gmail.com>

Description The package 'hrvm' faciliates estimation of parameters in Hierarchical Relevance Vector Machines (H-RVM) proposed by Srivastava et al. (2012). As a generalization of Tipping's (2001) relevance vector machine (RVM), H-RVM accommodates interactions between the input high-dimensional covariates from two sources (here: gene and miRNA expression) and induces dependence between them. H-RVM's learning algorithm is a special case of hierarchic kernel learning framework (HKL) of Girolami and Rogers (2005). This package is the translation of HKL's Matlab code to R that can be obtained from http://www.dcs.gla.ac.uk/~srogers/kernel_comb.htm. Please see the manual, help pages, and the website of Girolami and Rogers (2005) for appropriate citations. The HKL learning framework,its algorithm, and Matlab code are copyrighted to Girolami and Rogers (2005).

License GPL (>= 3)

LazyLoad yes

Depends MASS, VGAM, gtools, kernlab

Collate 'hierarchical-relevance-vector-machines.R' 'hrvm-package.R'

R topics documented:

hrvm-package 2
crossvalidateHrvm 6
cvFitErr 6
fitHrvm 7
fitSummary 8
sampleBeta 9
slog 10
updateAs 10
updateGamShapeRate 11
updatePhiShapeRate 12

Index **13**

`hrvm-package` *Implementation of the variational algorithm of Girolami and Rogers (2005) for fitting Hierarchical Relevance Vector Machines (H-RVM) For Integrating Multiplatform Genomic Data.*

Description

Implementation of the variational algorithm of Girolami and Rogers (2005) for fitting Hierarchical Relevance Vector Machines For Integrating Multiplatform Genomic Data.

Details

Package:	hrvm
Type:	Package
Version:	0.1
Date:	2012-08-16
License:	GPL (>= 3)
LazyLoad:	yes

`fitHrvm` is the workhorse function that fits the H-RVM using the variational algorithm of Girolami and Rogers (2005) as a special case of the Hierarchical Kernel Learning (HKL; see website). Srivastava et al. (2012) use the HKL algorithm of Girolami and Rogers (2005) and extend the relevance vector machine (RVM) of Tipping (2001) as hierarchical relevance vector machine (H-RVM).

Author(s)

Sanvesh Srivastava <sanvesh@gmail.com>

References

• M. Girolami and S. Rogers (2005). Hierarchic Bayesian Models for Kernel Learning. 22nd International Conference on Machine Learning (ICML 2005) 241–248. http://www.dcs.

gla.ac.uk/~srogers/kernel_comb.htm

- M. Tipping (2001). Sparse bayesian learning and the relevance vector machine. The Journal of Machine Learning Research, vol. 1, pp. 211–244, 2001.
- S. Srivastava, W. Wang, P. Zinn, R. R. Colen, and V. Baladandayuthapani (2012). Integrating Multi-Platform Genomic Data Using Hierarchical Bayesian Relevance Kernel Machines.

See Also

fitHrvm

Examples

```
## Set up the simulation
require(MASS)
require(kernlab)
set.seed(1234)

## True \beta
beta0 <- c(0.20, 0.36, 0.44)

## True \gamma
gamma0 <- 10.0

## True \phi
phi0 <- rep(5.0, 76)

## True \alpha
alpha0 <- mvrnorm(n = 1, mu = rep(0.0, 76), Sigma = diag(phi0^{-1}))

mu0 <- c(rep(0.5, 1000), rep (-0.5, 200))
sig0 <- matrix(0.0, nrow = 1200, ncol = 1200)
diag(sig0) <- rep(1.0, 1200)
sig0[lower.tri(sig0)] <- runif(1200 * 1199 / 2, -1, 1)

## patient data
geneMirnaExp <- mvrnorm(n = 75,
                        mu = mu0,
                        Sigma = sig0 %*% t(sig0)
                        )

genes0 <- geneMirnaExp[ , 1:1000]
mirna0 <- geneMirnaExp[ , 1001:1200]

## Column centered and standardized gene and mirna expression
stdgenes <- t(scale(t(genes0)))
stdmirna <- t(scale(t(mirna0)))

Krntmp <- matrix(0.0, nrow = 75, ncol = 1000 * 200)
for(i in 1:75) {
  Krntmp[i, ] <- kronecker(stdgenes[i, ], stdmirna[i, ])
}
```

```
## Standardized interaction
stdKtmp <- t(scale(t(Krntmp)))

## True Kernels in which gene, mirna, and their interaction will be embedded
kernGenes <- rbfdot(sigma = 1 )
kernMirnas <- rbfdot(sigma = 0.5)
kernInts <- rbfdot(sigma = 0.2 )

Kgenes <- rbind(rep(1, 75), kernelMatrix(kernGenes, stdgenes))
Kmirnas <- rbind(rep(1, 75), kernelMatrix(kernMirnas, stdmirna))
Kints <- rbind(rep(1, 75), kernelMatrix(kernInts, stdKtmp))

## True List of kernels
kList <- list(
           genes = Kgenes,
           mirnas = Kmirnas,
           ints = Kints
           )

## This is K_{\beta}^T calculated from the true kernels list
overallKernel <- diag(0.0, 75, 76)
for (k in 1:3) {
  overallKernel <- overallKernel + beta0[k] * t(kList[[k]])
}

## Simulate the tumor volume from these values
tvol <- mvrnorm(1,
                mu = overallKernel %*% alpha0,
                Sigma = 1 / gamma0 * diag(1.0, 75)
               )
## End simulation set up

## Train three RVMs to obtain the kernel parameter value for each of
## the three kernels.  That is, estimate \sigma_1, sigma_2, sigma_3.
genesKernSig2 <- rvm(stdgenes, tvol, kernel = "rbfdot")
mirnaKernSig2 <- rvm(stdmirna, tvol, kernel = "rbfdot")
intKernSig2 <- rvm(stdKtmp, tvol, kernel = "rbfdot")

## Estimated kernels, K_1, K_2, and K_3, used in the application of H-RVM
genesKern0 <- rbfdot(sigma = genesKernSig2@kernelf@kpar$sigma)
mirnaKern0 <- rbfdot(sigma = mirnaKernSig2@kernelf@kpar$sigma)
intKern0 <- rbfdot(sigma = intKernSig2@kernelf@kpar$sigma)

## The slightly abused version of K_1, K_2, and K_3.
genesKern <- rbind(rep(1, 75), kernelMatrix(genesKern0, stdgenes))
mirnaKern <- rbind(rep(1, 75), kernelMatrix(mirnaKern0, stdmirna))
intKern <- rbind(rep(1, 75), kernelMatrix(intKern0, stdKtmp))

## The list of kernels to be fed to fitHrvm function of hrvm package
kernList <- list(
              genes = genesKern,
              mirnas = mirnaKern,
              ints = intKern
```

```
              )

## The variational algorithm works with much fewer iterations (50)!
simRes <- fitHrvm(kernList, tvol, 1000, 50)
simRes$expBeta; simRes$expAlpha

## The result of fitting the H-RVM on the simulated data with 1000
## variational iterations.
simRes <- fitHrvm(kernList, tvol, 1000, 1000)
simRes$expBeta; simRes$expAlpha

## Compare the simulation results of H-RVM with other methods
## i. Comparison with penalized likelihood based method lasso:
library(glmnet)
genesMirnaLasso <- cv.glmnet(x = cbind(stdgenes, stdmirna), y = scale(tvol),
                             family = "gaussian", type.measure = "mse",
                             nlambda = 100, nfolds = 10, standardize = FALSE)
preds <- predict(genesMirnaLasso, cbind(stdgenes, stdmirna),
                 s = "lambda.1se", type = "response")

## ii. Gene RVM
geneRvm <- rvm(stdgenes, tvol, kernel = "rbfdot")

## iii. miRNA RVM
mirnaRvm <- rvm(stdmirna, tvol, kernel = "rbfdot")

## iv. interaction RVM
intRvm <- rvm(stdKtmp, tvol, kernel = "rbfdot")

## fit summary for the five models
fitSummary <- data.frame(geneRvm@fitted,
                         mirnaRvm@fitted,
                         intRvm@fitted,
                         simRes$kernel %*% simRes$expAlpha,
                         attr(scale(tvol), "scaled:scale") * preds
                          +  attr(scale(tvol), "scaled:center")
                         )
colnames(fitSummary) = c("Gene-RVM", "MiRNA-RVM", "Interaction-RVM", "H-RVM", "LASSO")

## summary of residuals for the five models
residSummary <- data.frame(tvol - geneRvm@fitted,
                           tvol - mirnaRvm@fitted,
                           tvol - intRvm@fitted,
                           tvol - simRes$kernel %*% simRes$expAlpha,
                  tvol - (attr(scale(tvol), "scaled:scale") * preds + attr(scale(tvol), "scaled:center"))
                          )
colnames(residSummary) = c("Gene-RVM", "MiRNA-RVM", "Interaction-RVM", "H-RVM", "LASSO")

library(lattice)
## lattice objects that can be used for checking fit for the five models
pG <- rfs(list(fitted.values = fitSummary[[1]], residuals = residSummary[[1]]), main = "Gene-RVM")
pM <- rfs(list(fitted.values = fitSummary[[2]], residuals = residSummary[[2]]), main = "MiRNA-RVM")
pI <- rfs(list(fitted.values = fitSummary[[3]], residuals = residSummary[[3]]), main = "Interaction-RVM")
```

```
pH <- rfs(list(fitted.values = fitSummary[[4]], residuals = residSummary[[4]]), main = "H-RVM")
pL <- rfs(list(fitted.values = fitSummary[[5]], residuals = residSummary[[5]]), main = "LASSO")
## print(pG); print(pM); print(pI); print(pH);print(pL)
```

`crossvalidateHrvm` *Calculate the H-RVM fit on training data*

Description

Calculate crossvalidation error between H-RVM and RVM results (use with caution; temporary solution!).

Usage

```
crossvalidateHrvm(kernels, y, nfolds = 10)
```

Arguments

`kernels`	training data kernel list for fitHrvm.
`y`	training response for fitHrvm.
`nfolds`	number of folds for crossvalidation.

Details

H-RVM fit on training data are compared against RVM fit from test data (although not great, but a meaningful way of sanity check).

References

http://stackoverflow.com/questions/7402313/generate-sets-for-cross-validation-in-r

---

`cvFitErr` *Compare the H-RVM fit on training data with RVM fit on test data*

Description

Calculate crossvalidation error between H-RVM and RVM results (use with caution; temporary solution!)

Usage

```
cvFitErr(hrvmFit, kernels, y)
```

Arguments

`hrvmFit`	object from the fitHrvm function.
`kernels`	list of kernels in the test data.
`y`	responses in the test data.

Details

H-RVM fit on training data are compared against RVM fit from test data (although not great, but a meaningful way of sanity check)

Value

list of fitted values and test error.

Author(s)

Sanvesh Srivastava (sanvesh@gmail.com)

`fitHrvm` *Fit H-RVM to estimate $E[\alpha]$ and $E[\beta]$.*

Description

Variational algorithm for fitting H-RVM using the variational algorithm of Girolami and Rogers (2005).

Usage

```
fitHrvm(kernels, y, beta.samp, max.it, pred.tol = 1e-05,
 hyperPars = list(phi = list(shape = 5, rate = 3), gamma = list(shape = 5, rate = 3), as = c(2, 3, 4)))
```

Arguments

`kernels`	list of length 3, representing source 1, 2, and interaction of source 1 and 2. The dimension of each kernel is (N + 1) $\times$ N, where N is the number of samples.
`y`	vector of length N.
`beta.samp`	number of samples to be used for estimating $E[\beta]$ and $E[\beta\beta^T]$
`max.it`	maximum iterations for the variational algorithm.
`pred.tol`	tolerance for parameter estimation and prediction.
`hyperPars`	the hyperparameters list for 3 parameters, γ, ϕ, and $\mathbf{a}$.

Details

Recodes the variational regression of Girolami and Rogers (2005) in R. This is translated Matlab code. For details see Section 4.1 of Girolami and Rogers (2005). Please see `http://www.dcs.gla.ac.uk/~srogers/kernel_comb.htm` for detailed examples and appropriate citations.

Value

list of $E[\alpha]$, $V[\alpha]$, $E[\alpha\alpha^T]$, $E[\beta]$, $E[\beta\beta^T]$, $E[\log\beta]$, $E[\gamma]$, $E[\phi]$, and `setUp` for fitting H-RVM.

Author(s)

Sanvesh Srivastava (sanvesh@gmail.com)

References

M. Girolami and S. Rogers (2005). Hierarchic Bayesian Models for Kernel Learning. 22nd International Conference on Machine Learning (ICML 2005) 241–248. http://www.dcs.gla.ac.uk/~srogers/kernel_comb.htm

---

`fitSummary` *Check residuals and predictions after H-RVM fit*

Description

Obtain residuals and predictions after H-RVM fit to be used in `rfs` plot of `lattice`.

Usage

```
fitSummary(res)
```

Arguments

`res`	object obtained from fitHrvm.

Value

list of fitted values and residuals.

Author(s)

Sanvesh Srivastava (sanvesh@gmail.com)

`sampleBeta` *Sample from the distribution of β and calculate $E[\beta]$, $E[\beta\beta\hat{}T]$, $E[\log\beta]$*

Description

Sample β and calculate $E[\beta]$, $E[\beta\beta^T]$, $E[\log\beta]$ as defined in Section 4.1 of Girolami and Rogers (2005).

Usage

```
sampleBeta(ndraws, expA, expGamma, omega, bs)
```

Arguments

`ndraws`	number of β to be drawn for calculating $E[\beta]$, $E[\beta\beta^T]$, $E[\log\beta]$.
`expA`	$(E[a_1]$, $E[a_2]$, $E[a_3])$.
`expGamma`	$E[\gamma]$.
`omega`	Ω as defined in Section 4.1 of Girolami and Rogers (2005).
`bs`	$\mathbf{b}$ as defined in Section 4.1 of Girolami and Rogers (2005).

Value

list of $E[\beta]$, $E[\beta\beta^T]$, $E[\log\beta]$.

Author(s)

Sanvesh Srivastava (sanvesh@gmail.com)

References

M. Girolami and S. Rogers (2005). Hierarchic Bayesian Models for Kernel Learning. 22nd International Conference on Machine Learning (ICML 2005) 241–248. http://www.dcs.gla.ac.uk/~srogers/kernel_comb.htm

`slog` *Safe log*

Description

Safe log of Girolami and Rogers (2005).

Usage

```
slog(x)
```

Arguments

x	vector

Details

Modifies the orignial log function for stability of H-RVM's variational algortihm.

Value

log(x) for x > 1e-10 and -10 for x <= 1e-10

Author(s)

Sanvesh Srivastava (sanvesh@gmail.com)

References

M. Girolami and S. Rogers (2005). Hierarchic Bayesian Models for Kernel Learning. 22nd International Conference on Machine Learning (ICML 2005) 241–248. http://www.dcs.gla.ac.uk/~srogers/kernel_comb.htm

`updateAs` *Update Dirichlet parameters (a_1, a_2, a_3) given $E[\beta]$.*

Description

Update parameters (a_1, a_2, a_3) of Dirichlet (a_1, a_2, a_3) given $E[\beta_1]$, $E[\beta_2]$, $E[\beta_3]$.

Usage

```
updateAs(betas)
```

Arguments

`as`	Current estimates of the parameters (a_1, a_2, a_3) of the Dirichlet distribution for β.
`logBetas`	$\log E[\beta]$.

Details

Use the type II maximum likelihood estimation procedure recommended by Girolami and Rogers (2005) to estimate the hyperparameters $\mathbf{a}$. Note that the Girolami and Rogers (2005) models them as random variables, but we estimate them here using VGAM package.

Value

updated parameter values of (a_1, a_2, a_3).

Author(s)

Sanvesh Srivastava (sanvesh@gmail.com)

References

- Thomas W. Yee and C. J. Wild (1996). Vector GeneralizedAdditive Models. Journal of Royal Statistical Society, Series B, 58(3), 481-493.
- Thomas W. Yee (2010). The VGAM Package for Categorical Data Analysis. Journal of Statistical Software, 32(10), 1-34. URL http://www.jstatsoft.org/v32/i10/.

`updateGamShapeRate` *Update the shape parameter c_γ of the Gamma distribution for γ*

Description

Update shape parameter c_γ of the Gamma distribution for γ.

Usage

```
updateGamShapeRate(expGamma, oldPar)
```

Arguments

`expGamma`	$E[\gamma]$.
`oldPar`	Current estimate of the shape parameter c_γ of the Gamma distribution for γ.

Details

Use the type II maximum likelihood estimation procedure recommended by Girolami and Rogers (2005) to estimate the shape parameter c_γ of the Gamma distribution for γ.

Value

updated shape parameter c_γ.

Author(s)

Sanvesh Srivastava (sanvesh@gmail.com)

References

- Thomas W. Yee and C. J. Wild (1996). Vector GeneralizedAdditive Models. Journal of Royal Statistical Society, Series B, 58(3), 481-493.
- Thomas W. Yee (2010). The VGAM Package for Categorical Data Analysis. Journal of Statistical Software, 32(10), 1-34. URL http://www.jstatsoft.org/v32/i10/.

`updatePhiShapeRate` *Update the shape parameter c_ϕ of the Gamma distribution for ϕ*

Description

Update shape parameter c_ϕ of the Gamma distribution for ϕ.

Usage

```
updatePhiShapeRate(expPhi, oldPar)
```

Arguments

`expPhi`	$E[\phi]$.
`oldPar`	Current estimate of shape parameter c_ϕ of the Gamma distribution for ϕ.

Details

Use the type II maximum likelihood estimation procedure recommended by Girolami and Rogers (2005) to estimate the shape parameter c_ϕ of the Gamma distribution for ϕ.

Value

updated shape parameter c_ϕ.

Author(s)

Sanvesh Srivastava (sanvesh@gmail.com)

References

- Thomas W. Yee and C. J. Wild (1996). Vector GeneralizedAdditive Models. Journal of Royal Statistical Society, Series B, 58(3), 481-493.
- Thomas W. Yee (2010). The VGAM Package for Categorical Data Analysis. Journal of Statistical Software, 32(10), 1-34. URL http://www.jstatsoft.org/v32/i10/.

Index

∗Topic **package**
hrvm-package, 2

crossvalidateHrvm, 6
cvFitErr, 6

fitHrvm, *2, 3*, *6*, 7, *7, 8*
fitSummary, 8

hrvm *(*hrvm-package*)*, 2
hrvm-package, 2

sampleBeta, 9
slog, 10

updateAs, 10
updateGamShapeRate, 11
updatePhiShapeRate, 12
